# Thyroid Hormones and Growth in Health and Disease

**DOI:** 10.4274/jcrpe.v3i2.11

**Published:** 2011-06-08

**Authors:** Ömer Tarım

**Affiliations:** 1 Uludağ University Faculty of Medicine, Division of Pediatric Endocrinology, Bursa, Turkey

**Keywords:** Thyroid, growth, children

## Abstract

Thyroid hormones regulate growth by several mechanisms. In addition to their negative feedback effect on the stimulatory hormones thyrotropin-releasing hormone (TRH) and thyrotropin (TSH), thyroid hormones also regulate their receptors in various physiological and pathological conditions. Up-regulation and down-regulation of the thyroid receptors fine-tune the biological effects exerted by the thyroid hormones. Interestingly, the deiodinase enzyme system is another intrinsic regulator of thyroid physiology that adjusts the availability of thyroid hormones to the tissues, which is essential for normal growth and development. Almost all chronic diseases of childhood impair growth and development. Every disease may have a unique mechanism to halt linear growth, but reduced serum concentration or diminished local availability of thyroid hormones seems to be a common pathway. Therefore, the effects of systemic diseases on thyroid physiology must be taken into consideration in the evaluation of growth retardation in affected children.

**Conflict of interest:**None declared.

## INTRODUCTION

**Introduction**

Thyroid hormones, along with insulin, growth hormone, glucocorticoids, insulin-like growth factor-1 (IGF-1) and other hormones, regulate body protein metabolism and, thereby, are closely linked to the processes involved in growth and development. This paper reviews the literature about the regulatory role of thyroid hormones in growth, in health and disease and concentrates on common clinical problems that can alter thyroid hormone status and, therefore, may play an important role in growth retardation observed in such conditions. 

**Role of the Thyroid Gland in Normal Growth** 

The first clues for a role of thyroid hormones in the regulation of cell proliferation were obtained from observations on amphibian metamorphosis. These observations have also revealed that other hormones including insulin, glucocorticoids, growth hormone  and prolactin participate in cell proliferation by antagonizing the effect of thyroxine (T4), at least in amphibians (1,2,3,4). During the process of development, apoptosis and cellular proliferation are balanced by this multihormonal mechanism, the major actor of which is triiodothyronine (T3) (5,6). Animal studies have demonstrated that T3 is a liver mitogen and promotes proliferation of hepatocytes, if given after partial hepatectomy and, its effect depends on the type and developmental state of the cell (7,8).  T3 also has positive effects on wound healing and on proliferation of cells, including cultured bovine thyroid cells, bone marrow pro-B cells, pancreatic acinar cells and renal proximal tubular epithelial cells (9,10,11,12,13). There are indications that T3 is required for branching morphogenesis and epithelial/mesenchymal differentiation of the lungs (14). Finally, T3 influences directly the linear growth, which may be via its stimulating effect on DNA synthesis in osteoblasts and in other cells (15). In contrast, T3 treatment has been reported to block proliferation and induce differentiation of oligodendrocyte progenitor cells, neuroblastoma N2a/b cells, and erythroid progenitors (16,17,18). Previous research has demonstrated that approximately 149 genes, including fibrinogen, transferrin, fibronectin (FN), androgen receptor (AR)-associated protein (ARA70), and dehydroepiandrosterone ulfotransferase family 1A member 2 (SULT2A1) genes, are positively regulated by T3. T3-target genes were investigated by microarray assay in hepatocellular carcinoma cell lines, and genes involved in metabolism, detoxification, signal transduction, cell adhesion and cell migration, as well as transcription factors, oncogenes, and the cell cycle were recognized to be up-regulated by treatment with T3 (2).  

T3 and its nuclear receptors modify expression of different genes/proteins involved in cell cycle control. This effect extends from growth factors [such as epidermal growth factor (EGF) and transforming growth factor (TGF)-b], to cell surface receptors (EGFR) as well as to proteins acting at the cell membrane level (Ras), various transcription factors (c-Fos, c-Myc, E2F1), cyclins, Cip/Kip family of cdk2 inhibitors, and p53 inhibitor Mdm2. The effect of thyroid hormones on these genes seems to vary with the type and developmental state of the cell and whether it is a normal or tumor cell (19).   The biological effects of T3 depend on various factors including amount of bioavailable hormone, levels of different thyroid receptor (TR) isoforms and of post-transcriptional modifications of TRs, type of their heterodimerization partners - retinoid X receptors (RXRs), interaction with co-repressors and co-activators , and on the structure of thyroid hormone response elements (TREs) in the target gene promoters  (20,21,22,23,24,25,26). The deiodinase (D) enzyme system is an important regulator of thyroid status via both pre- and posttranslational mechanisms that consequently play a significant role in regulating the availability of thyroid hormones to the tissues (27,28). As discussed in an extensive review by Germain et al (29), there are three types of D (D1, D2, D3) with different properties in terms of their activity in various tissues and their roles in states of hypothyroidism and hyperthyroidism.  The activities of D2 and D3 are designed to maintain local tissue T3 content as normal as possible in the face of altered serum hormone levels. In states of iodine deficiency and hypothyroidism, in order to maintain the available amount of T3 within the normal range, D2 activity is markedly up-regulated and D3 activity is decreased to increase the proportion of T3 formed locally and to lessen its degradation (30,31). Opposite changes in D2 and D3 activity occur in hyperthyroidism (32). Thyrotropin (TSH) stimulation of the thyroid gland in primary hypothyroidism results in increased D1 activity, which may serve to increase the conversion of T4 to T3 (33). The deiodinases are also important determinants of alterations in systemic thyroid hormone levels observed in illness and nutritional deficiency. Euthyroid sick syndrome or non-thyroidal illness  is considered as an adaptive response of the organism, although this definition is still controversial (34,35). Serum T4 and T3 levels are markedly decreased without a compensatory rise in the serum TSH level during severe illness and nutritional deficiency (36,37,38), leading to a significant decline in basal metabolic rate along with a decrease in protein and fat catabolism (39,40). Alterations in deiodinase activity have been postulated to be responsible for this adaptive suppression of the thyroid axis. Decreases in hepatic D1 activity and increases in hepatic and skeletal muscle D3 activity have been reported in this setting (36,41). On the other hand, more recent research has implied that alterations in D activity may be a consequence rather than a cause of the decrease in the serum T3 level (42,43). It was demonstrated that administration of supraphysiological amounts of T4 and/or T3 to rabbits with systemic illness was necessary to regulate the serum concentrations of these hormones, suggesting that enhanced hormonal degradation and/or excretion, rather than diminished thyroidal secretion or decreased T4 to T3 conversion, had a dominant role in the response to non-thyroidal illness (44). Recent research has suggested that induction of D3 activity in response to tissue injury and inflammation, due to hypoxic or oxidative stress, may influence healing or regenerative processes (45,46,47). However, more studies are needed to confirm these results.

**Thyroid and Growth in Disease**

Hypothyroidism is a well-known cause of growth retardation. Height prognosis in children with late-diagnosed congenital hypothyroidism is guarded. Although treatment leads to an initial catch-up growth spurt, prolonged hypothyroidism may result in compromised adult height (48). On the other hand, hyperthyroidism has been reported to accelerate growth in normal children and in patients with Turner syndrome (49). However, whether this temporary growth spurt increases final height is not known.

**Thyroid Physiology in Systemic Disease **     

Taking into account the important role of thyroid hormones in the regulation of growth, alterations in thyroid physiology must also be considered when evaluating the growth of a child with systemic disease. 

**Thyroid Function in Neuropsychiatric Disease**

Depression causes a blunted TSH response to thyrotropin-releasing hormone (TRH) stimulation and the expected nocturnal rise in TSH may be absent or diminished. The peripheral conversion of T4 to T3 is also decreased, a finding consistent with non-thyroidal illness or euthyroid sick syndrome (50). Lithium, used as a therapeutic agent in depression and psychosis, is notorious for causing hypothyroidism (51). Euthyroid sick syndrome may also occur in anorexia nervosa. The abnormal thyroid functions frequently include a low T3, high reverse T3 (rT3), normal or low T4, low-normal free T4 (fT4), and normal TSH (52). The TSH response to TRH and the iodine uptake on thyroid scan are often diminished. Even after a successful treatment of anorexia nervosa, the recovery period for thyroid hormones, particularly for T3, may be prolonged (53). Therefore, growth velocity of patients with anorexia nervosa may not normalize immediately after weight gain.  On the other hand, thyroid hormone treatment has also been advocated for patients with anorexia nervosa. It is doubtful that exogenous thyroid hormone can maintain normal growth in a state of energy deprivation. Long-term medications for various entities must be carefully assessed for their possible side effects on thyroid functions.  Methylphenidate, used for the treatment of attention deficit hyperactivity disorder, may cause modest reductions in serum T4 and TSH levels (1,54). However, it has also been reported that the serum concentrations of these hormones remain within normal range and that height, weight, body mass index (BMI), IGF-1, and IGF binding protein-3 (IGFBP-3) values are not significantly affected (54).  Many antiepileptic drugs also cause modest suppression of the hypothalamic-pituitary-thyroid axis, but clinical hypothyroidism is not reported (1,54). The long-term effects of such medication on linear growth remain to be elucidated. 

**Thyroid Functions in Hepatogastrointestinal Disease**

Since the liver is the major organ responsible for the metabolism and clearance of hormones, liver disease affects the serum concentration and activity of the hormones. Serum T4 concentration is increased in acute hepatitis, but clinical hyperthyroidism does not occur. This is due to the decreased clearance of T4 and to an increase in T4-binding globulin (TBG) levels as part of the acute-phase response to inflammation. The release of presynthesized TBG from damaged hepatocytes into the circulation is another cause of increment in TBG levels in these patients (55,56,57). Because T4 and TBG are increased simultaneously, hyperthyroidism is rarely a problem. Total and free T3 are usually decreased and rT3 - increased in acute hepatitis. However, T3, T4, and TSH may all be suppressed in fulminant hepatitis (58,59). Autoimmune liver disease may be associated with Hashimoto’s thyroiditis. Serum T3 is decreased in chronic liver disease due to the diminished activity of the hepatic enzyme 5-monodeiodinase, leading to reduced conversion of T4 to T3. Although total T4 is reduced, fT4 is usually normal (58). Certain medications used in liver disease may also alter thyroid function. Dexamethasone and propranolol inhibit D1 and contrast media used in cholangiography such as iopanoic acid and ipodate block the activity of both D1 and D2. All these medications reduce peripheral conversion of T4 to T3 and diminish clearance of T4, leading to increased T4 and decreased T3 concentrations. Consequently, serum rT3 and TSH are increased (60,61).

**Thyroid Functions in Renal Disease**

Similar to liver disease, peripheral conversion of T4 to T3 is diminished in chronic renal failure. Serum T4 and T3 levels are usually decreased, but unlike other systemic conditions that cause euthyroid sick syndrome, rT3 is usually normal.  The plasma TBG level is also usually normal, but TSH response to TRH and the thyroid response to TSH are reduced. Radioactive iodine uptake by the thyroid gland may be reduced, probably due to the increased serum concentration of free iodine which dilutes the radiolabeled iodine (62,63,64) 

**Thyroid Functions in Malnutrition and Obesity **

Serum TSH and TSH response to TRH have been reported to be diminished with acute fasting in adults (65,66). Adolescents with growth failure due to fear of obesity show a delayed TSH response to TRH (67). TSH response to TRH may be normal or delayed in patients with anorexia nervosa and nutritional dwarfing. Studies in both children and rats have demonstrated reduced T3 and increased  rT3 in this setting  (67,68,69). Serum T4, T3, fT4, and TSH concentrations were reported to be normal in exogenous obesity (70). However, more recent research findings indicate that serum fT4 is inversely and TSH is positively correlated with BMI, suggesting a state of subclinical hypothyroidism despite the presence of serum hormone concentrations within the normal range (71). 

## CONCLUSION

**Conclusion**

Although thyroid functions are affected by many systemic diseases, growth retardation in hese conditions is often multifactorial and it is difficult to attribute the retardation to thyroid dysfunction per se. The hypothalamic-pituitary-IGF-1 axis as well as the target tissues are also adversely affected in most of the conditions discussed above. Therefore, mechanisms that regulate growth during disease processes are complicated. Interactions within the endocrine system as well as the cross-talk between the immune, neuronal, and endocrine systems remain to be further elucidated to understand and manage the growth retardation associated with specific diseases.  
